# Supplementary material for: Lognormal distributions capture site-specific variability in enteric virus concentrations in wastewater
Source: Environ Sci (Camb). 2025 Oct 7;11(12):2973–85. doi: 10.1039/d5ew00286a (PMC12522195; doi:10.1039/d5ew00286a)
Supplement: EW-011-D5EW00286A-s001 [file EW-011-D5EW00286A-s001.pdf]

Supplementary Information (SI) for Environmental Science: Water Research & Technology.
This journal is © The Royal Society of Chemistry 2025

Supplementary information for:

Title: Lognormal distributions capture site-specific variations in enteric virus concentrations in wastewater

Authors: Chaojie Li[1], Tamar Kohn[1], Shotaro Torii[1], Htet Kyi Wynn[1], Alexander J Devaux[2], Charles Gan[2], Timothy R. Julian[2,3,4], Émile Sylvestre[5*]

[1] Laboratory of Environmental Virology, School of Architecture, Civil & Environmental Engineering (ENAC), École Polytechnique Fédérale de Lausanne, Lausanne, Switzerland

[2] Eawag, Swiss Federal Institute of Aquatic Science and Technology, Dübendorf, Switzerland

[3] Swiss Tropical and Public Health Institute, Allschwil, Switzerland

[4] University of Basel, Basel, Switzerland

[5] Sanitary Engineering, Delft University of Technology, Stevinweg 1, 2628 CN, Delft, the Netherlands

Correspondence: Correspondence and requests for materials should be addressed to E. Sylvestre (email: e.sylvestre@tudelft.nl)

Supplementary Information Contents:

S1 Quality control for the RT-dPCR assay
Quality control results for the enterovirus and norovirus duplex RT-dPCR assay.

S2 Additional results
Additional figures and tables not included in the main paper.

S1 Quality control for the EV / NoV duplex RT-dPCR assay

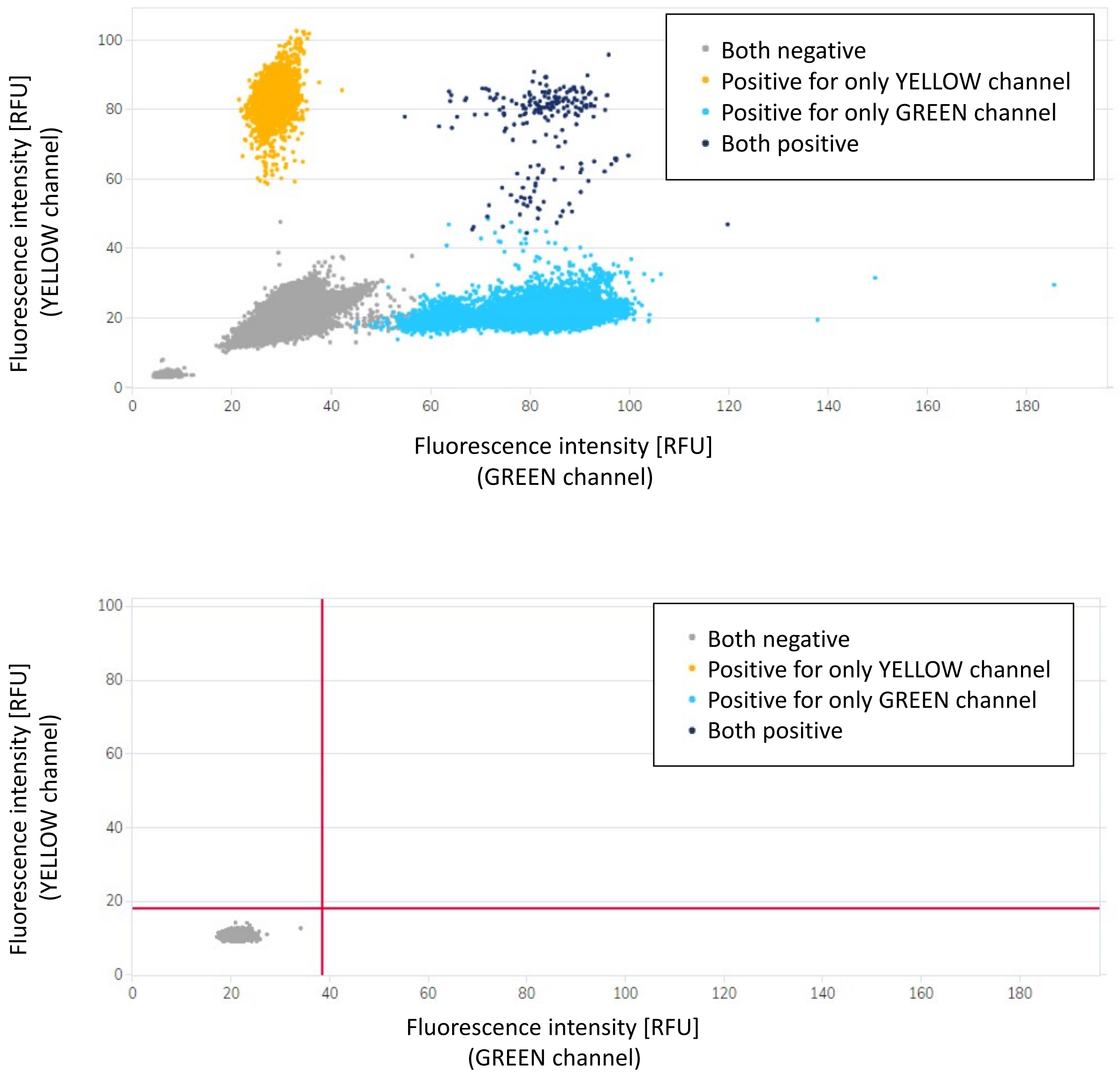

Fig. S1. Example fluorescence plots from the EV / NoV GII duplex RT-dPCR assay measured on the QIACuity One 2-plex device. EV is measured in the yellow channel, NoV GII is measured in the green channel. The plots include analysed wastewater samples collected between February 1 and May 2, 2021 (top) and those of negative controls (bottom). Red lines indicate automatically generated thresholds.

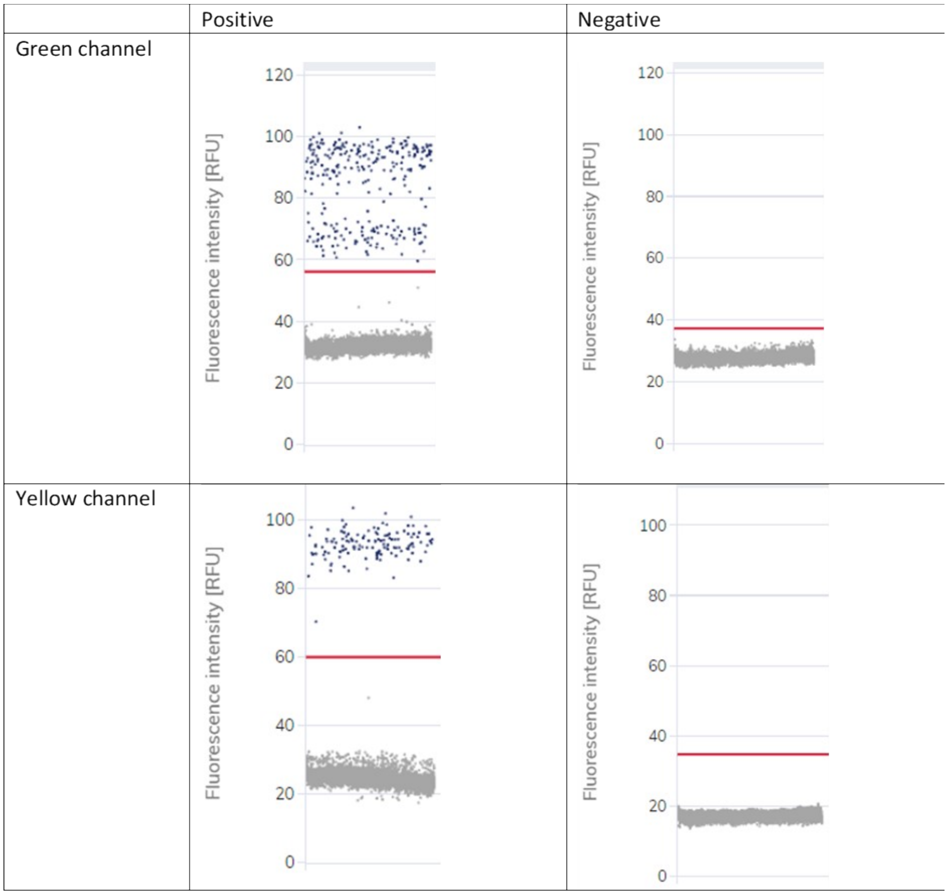

Fig. S2. Example outputs generated by the QIAcuity One 2-plex Device for a sample containing both EV (yellow channel) and NoV GII (green channel) and a negative control. Red lines show automatically generated thresholds between positive and negative partitions.

Correction factors

To determine the CFs, we conducted a structured comparison of Protocol 1 and Protocol 2 to evaluate whether the change in methods affected the quantification. In the period between November 10 and November 30, 2021, we processed all 21 collected samples using both protocols and independently determined their concentrations (see Figure S3 below). The concentrations obtained with Protocol 2 were 0.07 ± 0.20 log10 (1.17-fold) higher for EV and 0.42 ± 0.11 log10 (2.65-fold) higher for NoV GII compared to Protocol 1. A correction factor of 1.17 for EV and 2.65 for NoV GII was applied to the data before November 10th, obtained through protocol 1.

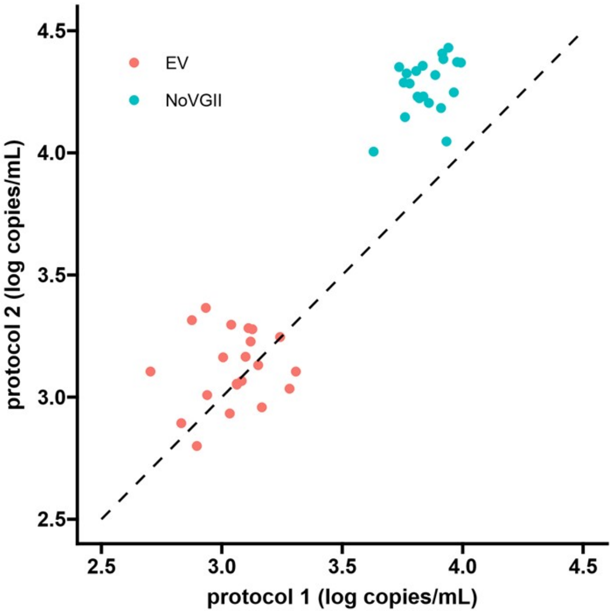

Fig. S3. Comparison of observed EV and NoV GII concentrations processed using Protocol 1 and Protocol 2. The dashed line represents the 1:1 relationship between concentrations obtained by the two protocols.

S2 Additional results

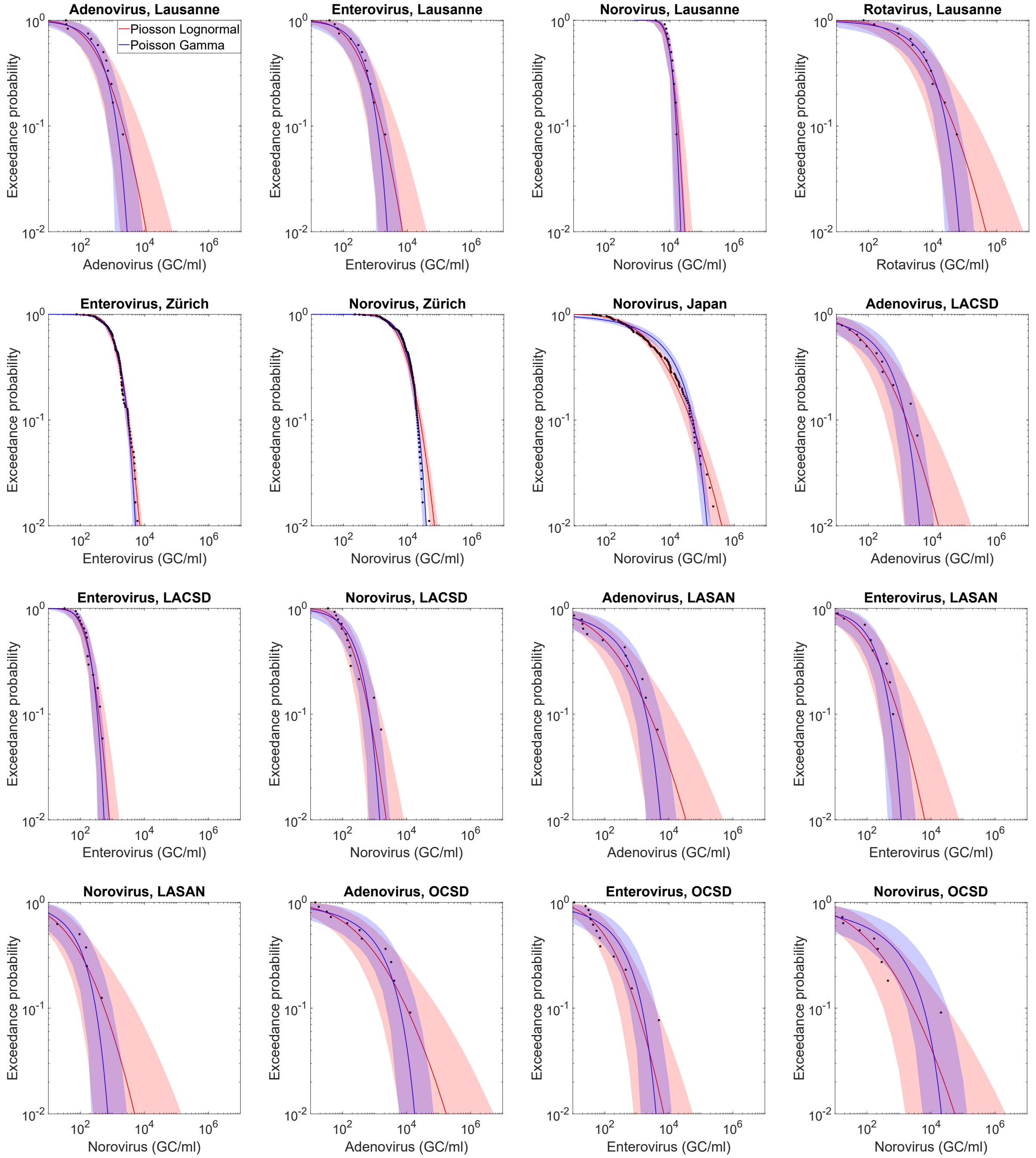

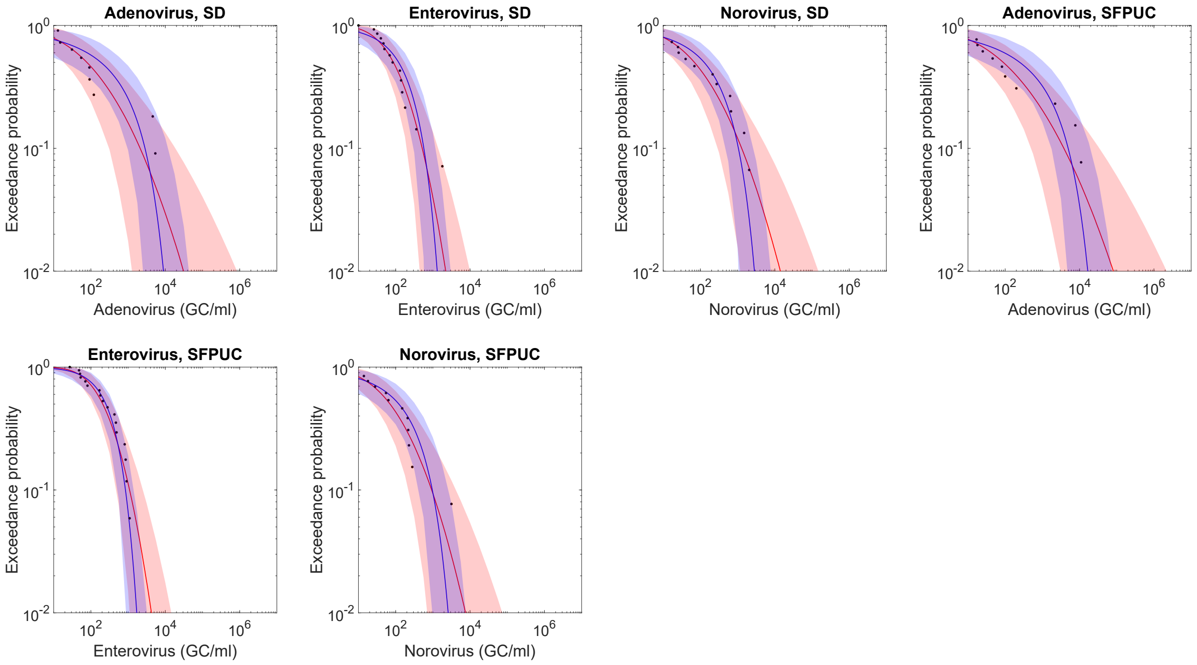

Fig. S4. Complementary cumulative distribution functions (CCDFs) of the mixed Poisson distributions of enteric virus concentrations for the eight wastewater treatment plants. Red curves represent PLN distributions and blue curves represent PGA distributions.

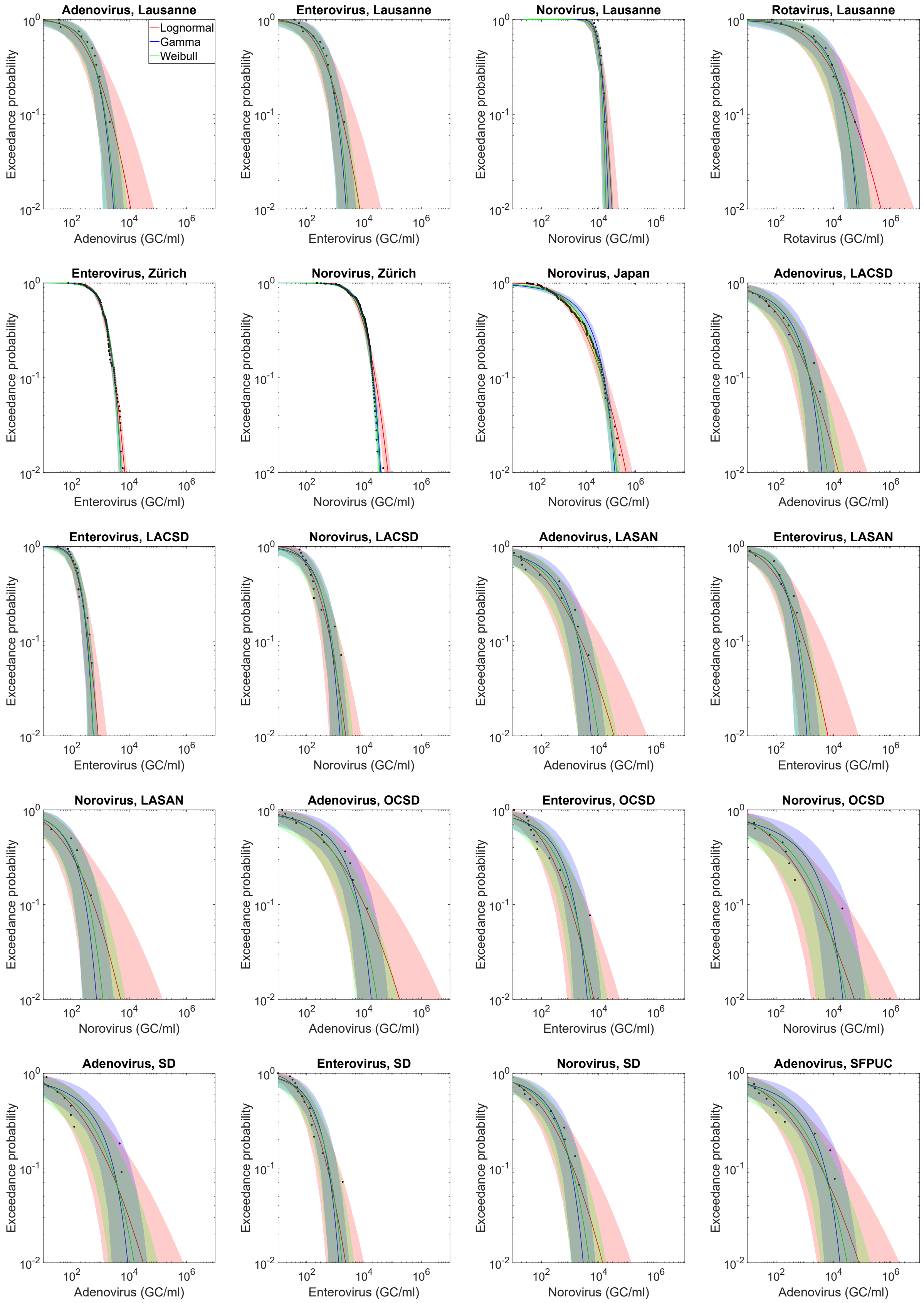
Adenovirus, Lausanne
Lognormal
Gamma
Weibull
Exceedance probability
Adenovirus (GC/ml)
Enterovirus, Lausanne
Enterovirus (GC/ml)
Norovirus, Lausanne
Norovirus (GC/ml)
Rotavirus, Lausanne
Rotavirus (GC/ml)
Enterovirus, Zürich
Enterovirus (GC/ml)
Norovirus, Zürich
Norovirus (GC/ml)
Norovirus, Japan
Norovirus (GC/ml)
Adenovirus, LACSD
Adenovirus (GC/ml)
Enterovirus, LACSD
Enterovirus (GC/ml)
Norovirus, LACSD
Norovirus (GC/ml)
Adenovirus, LASAN
Adenovirus (GC/ml)
Enterovirus, LASAN
Enterovirus (GC/ml)
Norovirus, LASAN
Norovirus (GC/ml)
Adenovirus, OCSD
Adenovirus (GC/ml)
Enterovirus, OCSD
Enterovirus (GC/ml)
Norovirus, OCSD
Norovirus (GC/ml)
Adenovirus, SD
Adenovirus (GC/ml)
Enterovirus, SD
Enterovirus (GC/ml)
Norovirus, SD
Norovirus (GC/ml)
Adenovirus, SFPUC
Adenovirus (GC/ml)

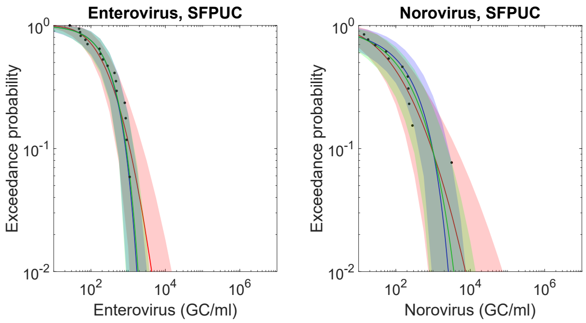

Fig. S5. Complementary cumulative distribution functions (CCDFs) of the continuous distributions of enteric virus concentrations for the eight wastewater treatment plants. Red curves represent lognormal distributions, blue curves represent gamma distributions, and green curves represent Weibull distributions.

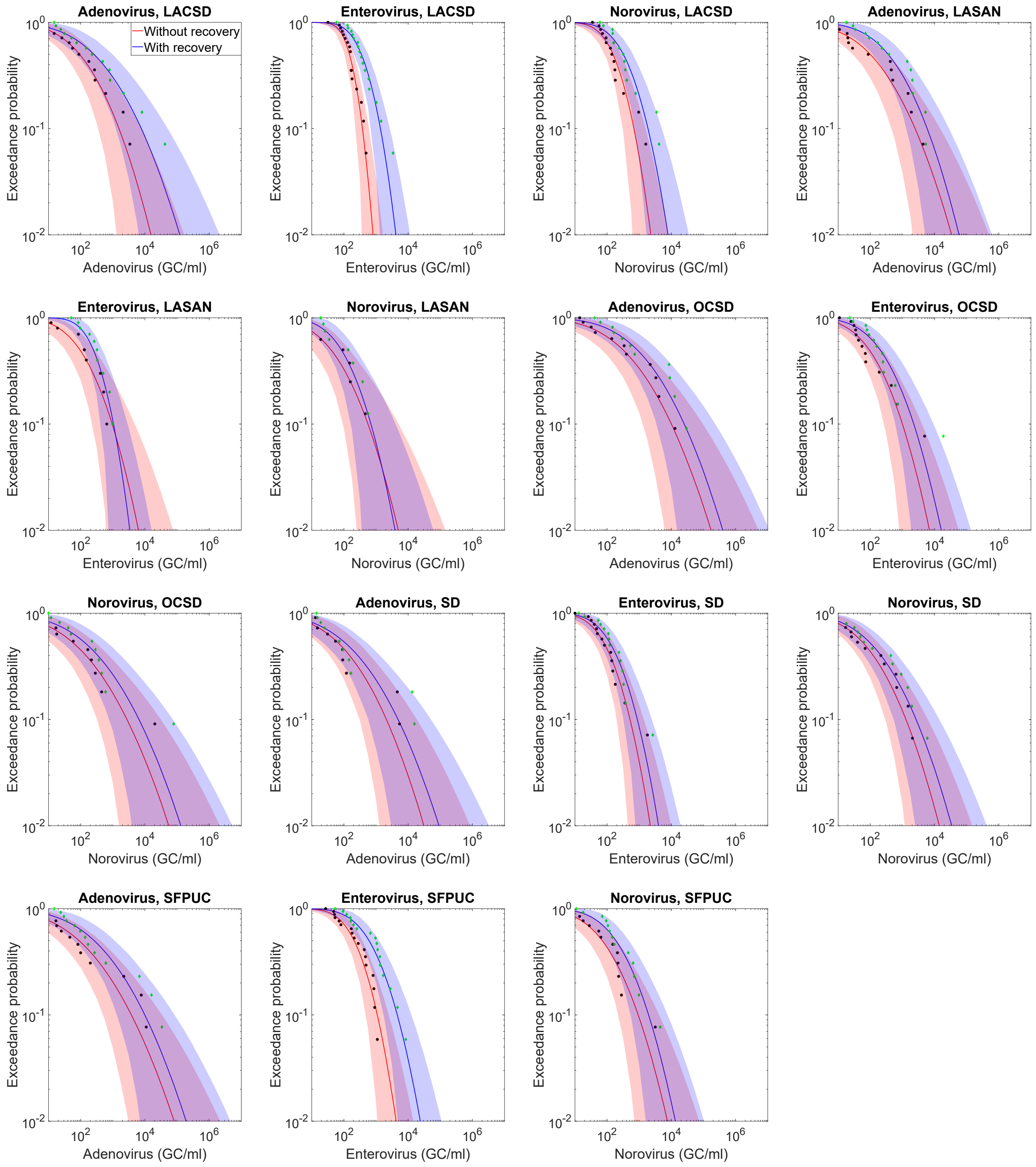

Fig. S6. Complementary cumulative distribution functions (CCDFs) of enteric virus concentrations with and without correcting for sample-specific recovery rates for the five wastewater treatment plants (WWTPs) from California, USA.

Table S1. mDIC of the mixed Poisson distributions and the DIC and AIC of the continuous distributions for wastewater treatment plants from Switzerland and Japan.

Dist.\Place	Lausanne, Switzerland				Zürich, Switzerland		Matsushima, Japan
	AD	ET	NR	RT	ET	NR	NR
**PLN**	**179**	**176**	**253**	**269**	**1632**	**2348**	**851**
PGA	181	182	247	280	1672	2352	922
Lognormal	*180*	*176*	*237*	*244*	*2968*	*2691*	*2648*
Gamma	*180*	*177*	*235*	*243*	*2969*	*3668*	*2684*
Weibull	*180*	*177*	*236*	*244*	*2977*	*3668*	*2663*
Lognormal	178	173	234	241	2967	3689	2651
Gamma	177	174	231	240	2974	3665	2686
Weibull	177	174	233	241	2976	3665	2664

[i] Dist. stands for distribution, AD stands for adenovirus, ET is enterovirus, NR is norovirus and RT represents rotavirus. The DIC values are in italic font while the AIC values are in normal font. The recommended distribution is in bold.

Table S2. mDIC of the mixed Poisson distributions and the DIC and AIC of the continuous distributions for wastewater treatment plants from California, USA.

Dist.\Place	LACSD			LASAN			OCSD			SD			SFPUC		
	AD	ET	NR	AD	ET	NR	AD	ET	NR	AD	ET	NR	AD	ET	NR
**PLN**	**271**	**207**	**209**	**223**	**130**	**93**	**203**	**205**	**169**	**173**	**217**	**200**	**216**	**237**	**197**
PGA	279	213	220	279	121	95	241	247	171	236	239	211	232	236	212
Lognormal	*382*	*444*	*379*	*389*	*270*	*205*	*332*	*351*	*301*	*301*	*369*	*403*	*363*	*474*	*346*
Gamma	*387*	*443*	*385*	*392*	*269*	*206*	*334*	*360*	*312*	*308*	*375*	*406*	*370*	*473*	*352*
Weibull	*419*	*509*	*423*	*414*	*295*	*211*	*347*	*380*	*321*	*320*	*404*	*420*	*384*	*525*	*372*
Lognormal	187	206	182	192	130	92	178	168	146	146	172	194	180	236	164
Gamma	192	206	188	196	128	92	180	178	156	154	172	196	188	236	170
Weibull	190	208	188	194	128	92	178	174	152	150	178	196	184	236	168

[ii] Dist. stands for distribution, AD stands for adenovirus, ET is enterovirus, NR is norovirus and RT represents rotavirus. The DIC values are in italic font while the AIC values are in normal font. The recommended distribution is in bold.
